# Supplementary material for: Neurogenesis during Brittle Star Arm Regeneration Is Characterised by a Conserved Set of Key Developmental Genes
Source: Biology (Basel). 2022 Sep 16;11(9):1360. doi: 10.3390/biology11091360 (PMC9495562; doi:10.3390/biology11091360)
Supplement: Supplementary file 1 [file biology-11-01360-s001.zip › biology-1889827-supplementary.pdf]

Supplementary material

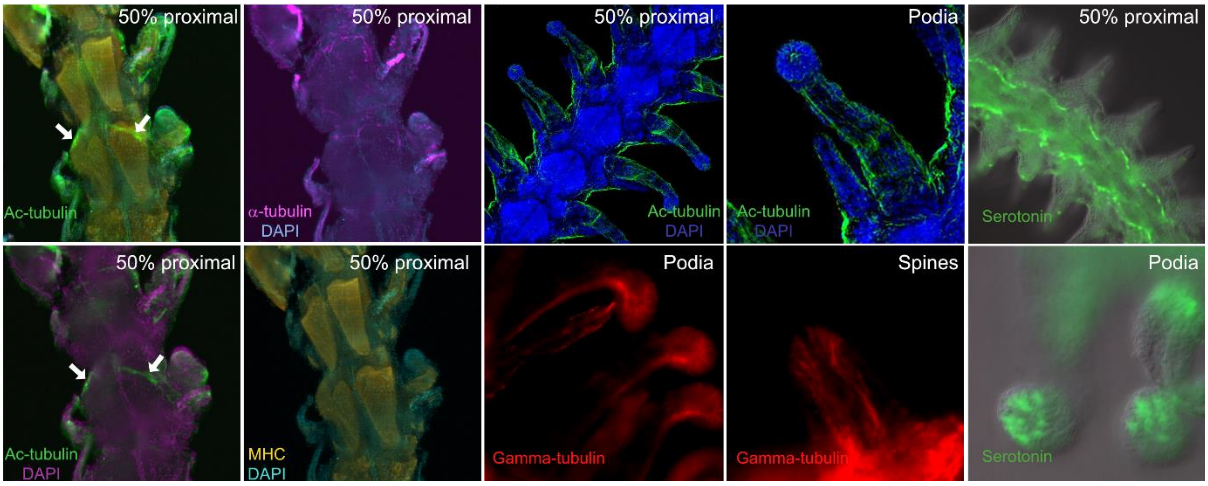

Figure S1: Nervous system antibody stainings in regenerating arms of *A. filiformis*. In the 50% fully differentiated proximal segments of a regenerating arm there is localized expression of MHC in intersegmental muscles, while alpha-tubulin and acetylated-tubulin antibodies stain the sensory neurons and relative neuronal projections from the podia and spine neurons. Arrows indicate proximal muscle nerves. Gamma-tubulin is restricted to peripheral nerve tracts lining the podia and spines. Serotonin-positive neuronal bodies can be seen forming clusters of cells running along the aboral side of the arm. A few single positive cells can be seen at the tips of the spines and clusters of positive serotonergic neurons are found at the tip of the podia.

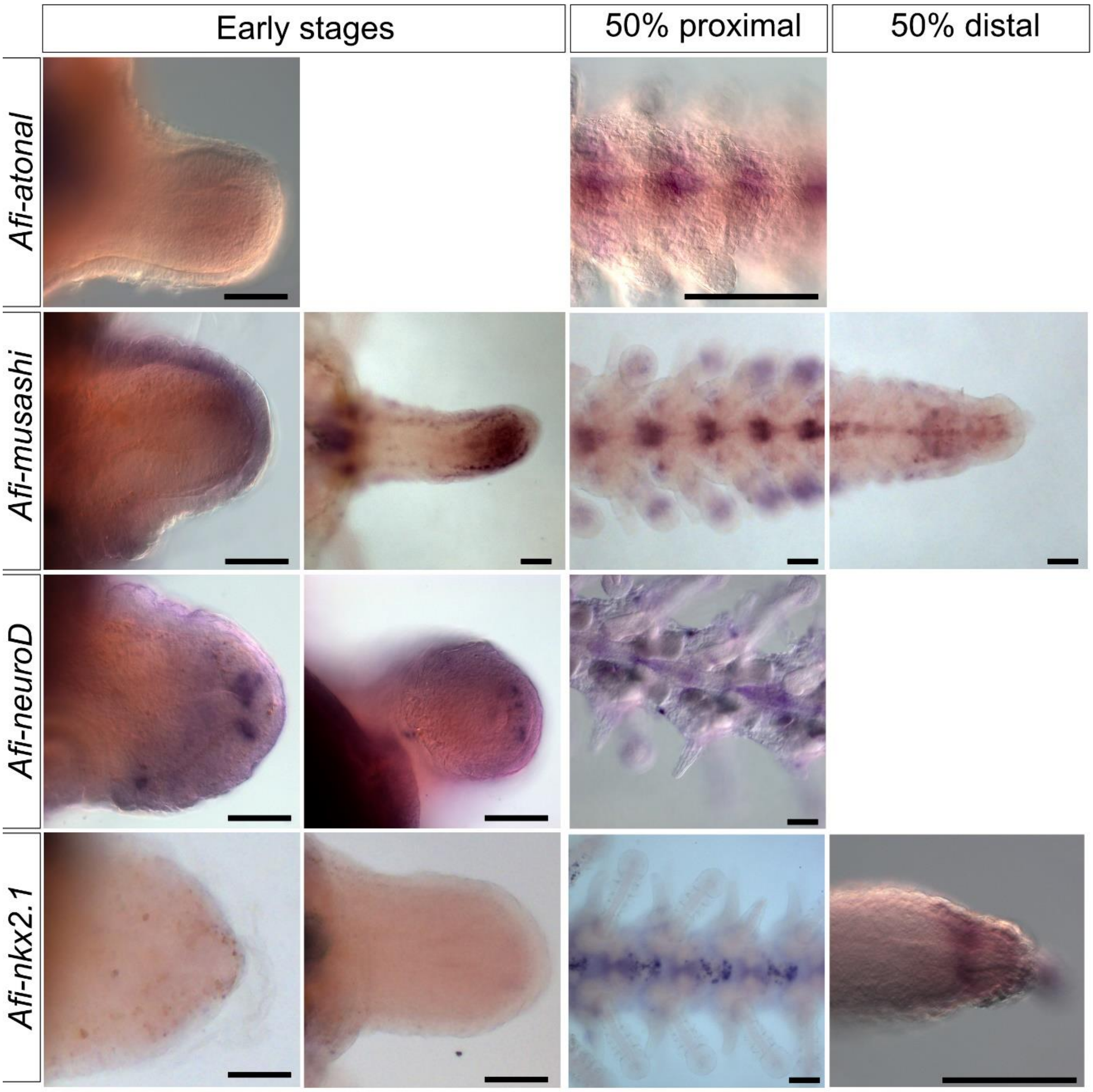

Fig S2: Whole mount in situ hybridization of *Afi-atonal, Afi-musashi, Afi-neuroD, Afi-nkx2.1* in *A. filiformis* at early (stage 2/3 and stage 4/5) and late (50% proximal and distal segments) stages of arm regeneration showing a variety of patterns within the different regions of the regenerating nervous system. *OV – oral view, OLV – oral-lateral view, LV – lateral view. Scale bar – 100 um.*

**Primary antibody**	**Cat #**	**Dilution**	**Antibody type**	**Epitope/Gene**	**Reference**
MHC	-	1:500	RP	Sp-MHC	Annunziata and Arnone 2014
SynB/1E11	-	1:500	MM	Sp-SynaptotagminB	Yoko Nakajima et al. 2004
alpha-Tubulin	Santa Cruz BioTech, sc-23948	1:500	MM	α-tubulin	
Ac-Tubulin	Sigma, T7451	1:500	MM	acetylated tubulin	
Gamma-Tubulin	Sigma, GTU 88	1:500	MM	γ-tubulin	
**Secondary antibody**	**Cat #**	**Dilution**	**Antibody type**	**Epitope/Gene**	**Reference**
Goat anti-Rabbit, Alexa Fluor 555	A-21428	1:1000			
Goat anti-Mouse, Alexa Fluor 488	A-11001	1:1000			
Goat anti-Rabbit, Alexa Fluor 633	A-21070	1:1000			
Goat anti-Mouse, Alexa Fluor 555	A-21422	1:1000			

Table S1: Primary and secondary antibodies used in this study and relative sources, MM – mouse monoclonal; RP – rabbit polyclonal

**Gene**	**Transciptome ID**	**Probe length (bp)**
Afi-elav	AfiCDS.id11028.tr61396	1167
Afi-soxC	AfiCDS.id33804.tr61034	919
Afi-soxB1	AfiCDS.id91348.tr60524	1209
Afi-six3	AfiCDS.id45608.tr3325	775
Afi-pax6	AfiCDS.id10654.tr45582	1335
Afi-neuroD	AfiCDS.id1482.tr19514	809
Afi-atonal	afiReg.id242420.tr108354	858
Afi-nkx2.1	AfiCDS.id21551.tr13724	1012
Afi-musashi	AfiCDS.id55115.tr52827	1245
Afi-soxB2	AfiCDS.id23770.tr24943	700

Table S2: List of probes used for WMISH. All cDNA fragments were cloned in pGEMT-easy vector and AS probes were transcribed using either T7 or Sp6 polimerase.

References:

Annunziata, Rossella, and Maria Ina Arnone. 2014. “A Dynamic Regulatory Network Explains ParaHox Gene Control of Gut Patterning in the Sea Urchin.” *Development (Cambridge, England)* 141 (12): 2462–72. https://doi.org/10.1242/dev.105775.

Yoko Nakajima, Hiroyuki Kaneko, Greg Murray, and Robert D. Burke. 2004. “Divergent Patterns of Neural Development in Larval Echinoids and Asteroids.” *Evolution & Development* 6: 95–104.
